# Airway Management for Massive Anterior Mediastinal Tumor Resection in an Infant: A Strategy Involving Spontaneous Breathing-Preserving Endotracheal Intubation under Intravenous Anesthesia

**DOI:** 10.1155/2024/1727612

**Published:** 2024-05-27

**Authors:** Hiromi Matsuda, Ei Ito, Akiko Katsuike, Hirotsugu Okamoto

**Affiliations:** Department of Anesthesiology, Kitasato University School of Medicine, 1-15-1 Kitasato, Kanagawa 252-0375, Japan

## Abstract

Tracheal intubation under sedation in uncooperative infants is challenging. The case of a 4-month-old infant with a massive anterior mediastinal tumor and upper respiratory tract symptoms, for whom effective preoxygenation was provided with a high-flow nasal cannula (HFNC), allowing for safe tracheal intubation in combination with a supraglottic device and local anesthetic, is reported. With careful planning of anesthesia and creative problem solving, airway management for anterior mediastinal tumors can be performed safely with the selection of an appropriate airway device. This may be a good airway management strategy for infants with mediastinal tumors or who may be expected to have ventilation difficulties.

## 1. Introduction

Preoxygenation is difficult in pediatric patients due to lack of cooperation, and even in cases of difficult airways, conscious intubation is extremely difficult and dangerous, so it is rarely performed. However, in cases in which ventilation difficulties are anticipated before general anesthesia, endotracheal intubation may be necessary under sedation while preserving spontaneous breathing. This report describes the safe performance of tracheal intubation without causing pain or the cough reflex in the patient while preserving spontaneous breathing during anesthesia induction for anterior mediastinal tumor resection in an infant with upper respiratory symptoms.

## 2. Case Presentation

**Figure 1:**
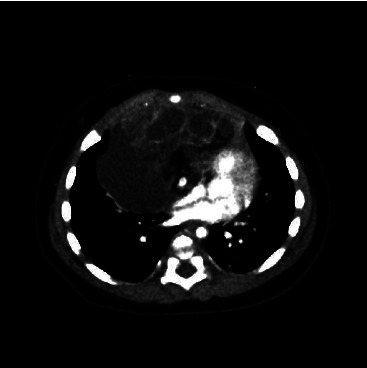
Chest computed tomography image. A tumorous lesion is seen in the anterior mediastinum, with compression of the heart and distal area of the carina.

A healthy 4-month-old male infant (height 60 cm and weight 7.4 kg) was scheduled for removal of a massive anterior mediastinal tumor. He had been wheezing for several weeks and was diagnosed with bronchitis at the clinic and prescribed an expectorant, but cyanosis was gradually observed. Transcutaneous oxygen saturation (SPO_2_) was 60% in the supine position and 99% in Fowler's position and crying decreased SPO_2_ and worsened wheezing. Chest computed tomography (CT) showed a tumorous lesion in the anterior mediastinum, with compression of the heart and the distal area of the carina (Figure 1). The anterior mediastinum tumorectomy was scheduled for one week later, and the patient was managed in the pediatric intensive care unit with a head-up position of over 30° and high-flow nasal cannula (HFNC) therapy (FiO_2_ 30%, 15 L) until then.

## 3. Airway Management

General anesthesia was administered with rescue equipment including extracorporeal membrane oxygenation (ECMO) on standby.

The patient was placed in the ramp position and sedated with atropine 0.01 mg/kg, midazolam 0.2 mg/kg, pethidine 1 mg/kg, and propofol 4–10 mg/kg/h while oxygenation was continued with HFNC. An i-gel (size 1.5, Workingham, UK) was inserted, and spontaneous breathing was confirmed after sedation (Video 1, https://youtu.be/YfKaeT1dIMY). Local anesthesia was performed using an epidural catheter (Perifix ONE Catheter, B. Braun, Melsungen, Germany) with a side hole cut in advance from the forceps port of the bronchoscope (BF-XP190, Olympus, Tokyo, Japan) via the i-gel (Video 2, https://youtu.be/sC-2pncx8CA). For local anesthesia, 1 mL of a 4-fold dilution of lidocaine (4%, SANDOZ, Tokyo, Japan) was sprayed onto the vocal cords and trachea. After confirming that the vocal cord reflex had disappeared, the i-gel was removed, and a 3.5-mm Microcuff endotracheal tube (Avanos, Alpharetta, GA, USA) was used for intubation under videolaryngoscopic guidance (McGRATH, MAC1, Covidien, Heerlen, The Netherlands)(Video 3, https://youtu.be/ZLYGYyfWubk).

After tracheal intubation, it was confirmed that ventilation was possible with pressure support of 25–30 cmH_2_O, with anesthesia maintained with 0.8% sevoflurane and remifentanil 0.1*γ*, and spontaneous breathing preserved with manual ventilation until the sternal incision. After the sternum was incised, the tidal volume was maintained sufficiently, and controlled ventilation could be used. From induction of anesthesia to sternotomy, the patient was managed with FiO_2_ 100%, but after sternotomy, FiO_2_ was gradually lowered to 40%. No muscle relaxants were used from the induction of anesthesia to the end of the surgery.

On postoperative day (POD) one, the patient was extubated after the trachea was observed using a bronchoscope in the PICU. Due to slight evidence of tracheomalacia, HFNC (FiO_2_ 30%, 15 L) was used until POD2. The patient was stable for discharge home on POD8.

## 4. Discussion

Pediatric anterior mediastinal tumors are characterized by a high risk of cardiopulmonary complications [1, 2], and general anesthesia with use of muscle relaxants should be avoided [3]. Because most anterior mediastinal tumors showing rapid onset of symptoms in children are lymphomas or leukemias [4, 5], chemotherapy and radiation therapy could be considered first-line treatment [6]. It is important to discuss the indication for the surgical procedure under general anesthesia with multiple specialists [4]. In the present case, total tumor resection was scheduled, and the definitive diagnosis of this case was immature teratoma of the thymus.

Even with endotracheal intubation, especially in the supine position, the gravity of the chest wall and the tumor reduce lung compliance, and there is a high risk that ventilation will become impossible due to the loss of bronchial muscle tone. Moreover, since the difficult airway guidelines are ineffective in this critical situation, a spontaneous breathing technique is most commonly advocated [1].

There is no cause-and-effect relationship established between anesthetic technique and complications [2], and there is no established method for inducing general anesthesia and conducting airway management in patients with anterior mediastinal tumors presenting with orthopnea, wheezing, and cyanosis. Generally, endotracheal intubation that preserves spontaneous breathing in pediatric patients is difficult and is, therefore, not often selected. In the present case, by selecting an appropriate airway device, careful anesthesia planning, and with creative problem solving, it was possible to safely manage the airway during anterior mediastinal tumor resection without any complications.

Even though there are no definitive studies comparing the efficacy of the airway management process between facemasks and HFNC for pediatric cases, HFNC is attracting increased attention and may provide safe preoxygenation [7]. HFNC can be a feasible option for children who are noncompliant to the tight-fitting facemask [8] during the induction of general anesthesia and highly susceptible to rapid desaturation. Since supraglottic airway devices are now being used in children as a first-choice airway device and as a conduit for tracheal intubation [9], the i-gel was selected in the present case as a route to facilitate easy provision of local anesthesia under spontaneous breathing to the upper airway. Moreover, the combination of an epidural catheter made it possible to effectively administer local anesthesia to the intended area; in this case, the vocal cord and the trachea, with a small volume of fluid.

In our view, the method presented is effective and can be used as a strategy for tracheal intubation in uncooperative pediatric patients for whom there are concerns about the use of muscle relaxants or who are expected to experience difficult ventilation.

## Data Availability

Due to the nature of this report, supporting data are not available.

## Supplementary Materials

Supplementary Materials Video 1: supraglottic airway device insertion. After intravenous sedation, i-gel is inserted, and spontaneous breathing is confirmed. Video 2: the local anesthesia method. Local anesthesia is performed using an epidural catheter via the i-gel. 1 mL of lidocaine is sprayed onto the vocal cords and trachea. The patient was coughing. Video 3: endotracheal intubation. The i-gel is removed, followed by intubation with a 3.5-mm endotracheal tube under videolaryngoscopic guidance.
