# Supplementary material for: Airway Management for Massive Anterior Mediastinal Tumor Resection in an Infant: A Strategy Involving Spontaneous Breathing-Preserving Endotracheal Intubation under Intravenous Anesthesia
Source: Case Rep Pediatr. 2024 May 27;2024:1727612. doi: 10.1155/2024/1727612 (PMC11149395; doi:10.1155/2024/1727612)
Supplement: Supplementary Materials — Video 1: supraglottic airway device insertion. After intravenous sedation, i-gel is inserted, and spontaneous breathing is confirmed. Video 2: the local anesthesia method. Local anesthesia is performed using an epidural catheter via the i-gel. 1 mL of lidocaine is sprayed onto the vocal cords and trachea. The patient was coughing. Video 3: endotracheal intubation. The i-gel is removed, followed by intubation with a 3.5-mm endotracheal tube under videolaryngoscopic guidance. [file 1727612.f1.docx]

Supplementary Material :

Video legends

Video 1: Supraglottic airway device insertion

After intravenous sedation, i-gel is inserted, and spontaneous breathing is confirmed.

[
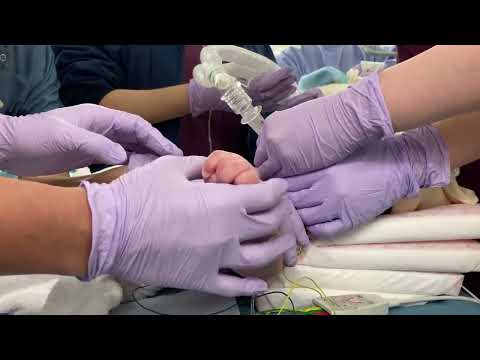
](https://www.youtube.com/embed/YfKaeT1dIMY?feature=oembed)

Video 2: The local anesthesia method

Local anesthesia is performed using an epidural catheter via the i-gel. 1 mL of lidocaine is sprayed onto the vocal cords and trachea. The patient was coughing.

[
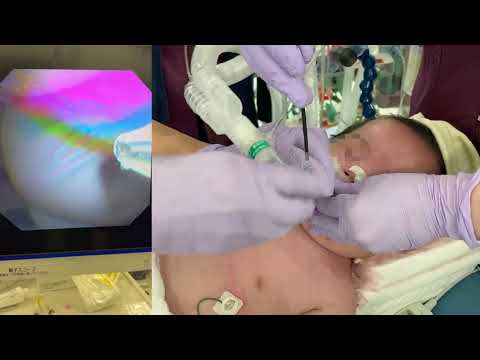
](https://www.youtube.com/embed/sC-2pncx8CA?feature=oembed)

Video 3: Endotracheal intubation

The i-gel is removed, followed by intubation with a 3.5-mm endotracheal tube under videolaryngoscopic guidance.

[
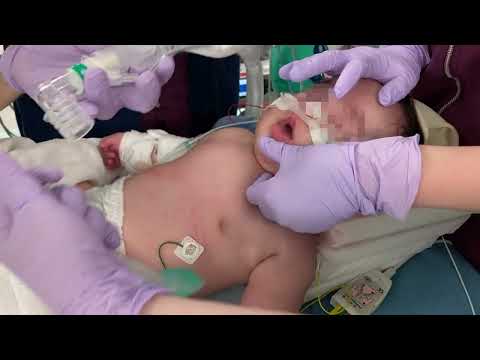
](https://www.youtube.com/embed/ZLYGYyfWubk?feature=oembed)
